# Prevalence of morbidity symptoms among pregnant and postpartum women receiving different nutrient supplements in Ghana and Malawi: A secondary outcome analysis of two randomised controlled trials

**DOI:** 10.1111/mcn.13501

**Published:** 2023-04-06

**Authors:** Seth Adu‐Afarwuah, Charles D. Arnold, Anna Lartey, Harriet Okronipa, Kenneth Maleta, Per Ashorn, Ulla Ashorn, Kathryn G. Dewey

**Affiliations:** ^1^ Department of Nutrition and Food Science University of Ghana Legon Ghana; ^2^ Institute for Global Nutrition, Department of Nutrition University of California Davis California USA; ^3^ Department of Nutritional Sciences Oklahoma State University Stillwater Oklahoma USA; ^4^ Department of Public Health, School of Public Health and Family Medicine University of Malawi College of Medicine Blantyre Malawi; ^5^ Faculty of Medicine and Health Technology, Center for Child, Adolescent and Maternal Health Research Tampere University Tampere Finland; ^6^ Department of Pediatrics Tampere University Hospital Tampere Finland

**Keywords:** common morbidity symptoms, maternal morbidity, multiple micronutrient supplements, small‐quantity lipid‐based nutrient supplements

## Abstract

Little is known about the impact of small‐quantity lipid‐based nutrient supplements (SQ‐LNSs) on maternal morbidity. This secondary outcome analysis aimed to compare morbidity symptoms among women in two trials evaluating the efficacy of SQ‐LNSs. From enrolment (≤20‐week gestation) to 6 months postpartum, Ghanaian (*n* = 1320) and Malawian (*n* = 1391) women were assigned to consume daily: 60 mg iron and 400 µg folic acid until childbirth and placebo thereafter (iron and folic acid [IFA] group); or multiple micronutrients (MMN); or 20 g/day SQ‐LNSs. Within country, we used repeated measures logistic regression and analysis of variance models to compare group differences in the period prevalence and percentage of days of monitoring when women had fever, gastrointestinal, reproductive, and respiratory symptoms during the second and third trimesters of pregnancy (*n* ~ 1243 in Ghana, 1200 in Malawi) and 0–3 and 3–6 months postpartum (*n* ~ 1212 in Ghana, 730 in Malawi). Most outcomes did not differ significantly among groups, with the following exceptions: in Ghana, overall, the prevalence of vomiting was lower in the LNS (21.5%) than MMN (25.6%) group, with the IFA group (23.2%) in‐between (*p* = 0.046); mean ± SD percentage of days with nausea was greater in the LNS (3.5 ± 10.3) and MMN (3.3 ± 10.4) groups than the IFA (2.7 ± 8.3) group (*p* = 0.002). In Malawi, during 3–6 month postpartum, the prevalence of severe diarrhoea was greater in the LNS (8.1%) than the MMN (2.9%) group, with IFA (4.6%) in‐between, *p* = 0.041). We conclude that the type of nutrient supplement received during pregnancy and lactation generally does not influence morbidity symptoms in these settings. Clinicaltrials.gov identifiers: NCT00970866; NCT01239693.

## Key messages

- Ghanaian and Malawian women assigned to receive iron and folic acid (pregnancy only) or multiple micronutrients (pregnancy and lactation) or small‐quantity lipid‐based nutrient supplements (SQ‐LNSs) (pregnancy and lactation) generally did not differ in morbidity outcomes during the second and third trimesters of pregnancy and 0–3 and 3–6 months lactation.
- The lack of group differences in most of the morbidity outcomes corroborates previous results of no significant group differences in the percentage of women who experienced severe adverse events or mean concentrations of two biomarkers of inflammation (C‐reactive protein and alpha‐1 glycoprotein) or percentage of women with elevated concentrations of the biomarkers.
- Overall, there was no consistent evidence that SQ‐LNS or multiple micronutrients increased or decreased maternal morbidity during pregnancy or lactation, compared to iron and folic acid.

## 1 INTRODUCTION

Common morbidity symptoms such as fever, poor appetite, nausea and vomiting are experienced by many pregnant and lactating women (Regodón Wallin et al., 2020) and can have debilitating consequences such as reduced quality of life, depressive symptoms and malnutrition (Regodón Wallin et al., 2020; Temming et al., 2014). However, these symptoms have generally received little research attention (Parikh et al., 2021; Roos‐Hesselink et al., 2019; Vogel et al., 2021).

Low nutrient status during pregnancy and lactation could contribute to the occurrence and severity of morbidity symptoms (Papathakis et al., 2016; Wu, Imhoff‐Kunsch, & Girard, 2012). Current estimates suggest that 80% of nonpregnant women 15–49 years of age in Sub‐Saharan Africa (161 million) have deficiencies of at least one of three core micronutrients, including iron, zinc and folate (Stevens et al., 2022) attributed primarily to poor access to nutritious foods (Darnton‐Hill & Mkparu, 2015; Lee et al., 2013). Similar estimates are not available for pregnant and lactating women (Stevens et al., 2022), but the prevalence of deficiency could be even higher due to increased physiological requirements during those periods (Adu‐Afarwuah, Lartey, & Dewey, 2017).

Small‐quantity (20 g/day) lipid‐based nutrient supplements (SQ‐LNSs) were developed for pregnant and lactating women by collaborators of the International Lipid‐based Nutrient Supplements (iLiNS) Project (Arimond et al., 2015) to add to the menu of interventions for addressing poor access to nutritious foods in low‐income settings (Das et al., 2018). Besides micronutrients, SQ‐LNSs provide high‐quality protein and essential fatty acids (EFAs) linoleic acid and α‐linolenic acid, which are not usually included in multiple micronutrient supplements.

We previously reported pregnancy outcomes from the iLiNS‐DYAD trials in Ghana (Adu‐Afarwuah et al., 2015) and Malawi (Ashorn, Alho, Ashorn, Cheung, Dewey, Harjunmaa, et al., 2015) that evaluated the efficacy of maternal SQ‐LNSs. In Ghana, maternal SQ‐LNS had a positive impact on several outcomes including greater foetal growth (especially among primiparous women) (Adu‐Afarwuah et al., 2015), decreased prevalence of low gestational weight gain (Adu‐Afarwuah, Lartey & Okronipa, Ashorn, et al., 2017), and increased likelihood of adequate iodine status (Adu‐Afarwuah et al., 2018). In Malawi (Ashorn, Alho, Ashorn, Cheung, Dewey, Harjunmaa, et al., 2015), results for foetal growth were less conclusive but generally consistent with those from Ghana (Adu‐Afarwuah et al., 2015). Evaluating the impact of SQ‐LNS consumption on women's morbidity is important for identifying additional potential benefits of this intervention, as well as potential risks.

The aim of this secondary outcome analysis was to compare morbidity symptoms among women enrolled in the iLiNS‐DYAD trials in Ghana and Malawi. We hypothesised that within country, the three treatment groups would differ in the prevalence and mean percentage of days of monitoring when women had morbidity symptoms during pregnancy and lactation.

## 2 METHODS

### 2.1 Study design, setting and participants

As described previously, the trials in Ghana (Adu‐Afarwuah et al., 2015, 2016) and Malawi (Ashorn, Alho, Ashorn, Cheung, Dewey, Gondwe, et al., 2015; Ashorn, Alho, Ashorn, Cheung, Dewey, Harjunmaa, et al., 2015) were partially double‐blind, parallel, individually randomised, controlled trials with three equal‐size groups.

In brief, we conducted the Ghana trial (ClinicalTrials.org Identifier: NCT00970866) in the Somanya‐Odumase‐Kpong area, a semi‐urban setting about 70 km north of Accra. At the time of the study, the inhabitants were mainly subsistence farmers and petty traders; by Ghanaian standards, most had low socioeconomic status. Adult literacy was ~44%. The staple diets consisted of maize, cassava, plantain, rice, fish and leafy vegetables; mango and watermelon consumption was seasonal. Health services were provided mainly by two hospitals, one polyclinic (providing more services than a clinic but not to a hospital status) and one clinic run by the government. Between December 2009 and December 2011, women on routine attendance to antenatal clinics in the four health facilities were eligible if they were ≥18 years old, ≤20 weeks pregnant, and their antenatal cards contained the information necessary for determining eligibility. We included women ≥18 years of age because the legal age of adulthood was 18 years, and the prevalence of pregnancy among girls <18 years at the study site was relatively low. Exclusion criteria were: intention to move out of the area during the period of the intervention; milk or peanut allergy; unwillingness to receive field workers or take study supplement; gestational age (GA) > 20 weeks before completion of enrolment; antenatal card indicated HIV infection, asthma, epilepsy, tuberculosis, or malignant disease; known to have previously enrolled in the same trial; or currently taking part in another clinical trial. The trial was approved by the ethics committees of the University of California, Davis; the Ghana Health Service; and the University of Ghana Noguchi Memorial Institute for Medical Research.

In Malawi, we conducted the trial (ClinicalTrials.org Identifier NCT01239693) in the Mangochi District, a predominantly rural area south of the country. The main sources of livelihood in the area were subsistence farming and fishing, and the diets consisted predominantly of cereals, mainly corn, with relatively small amounts of roots, tubers, fish, fruit and legumes (Ndekha et al., 2000). Most inhabitants had low socioeconomic status. Between February 2011 and August 2012, women on routine attendance to antenatal clinics at four main health facilities were eligible if they lived in the Malindi Hospital or Lungwena Health Centre catchment areas in the Mangochi District, were ≤20 weeks pregnant by ultrasound assessment, were available during the period of the study and gave informed consent. Exclusion criteria were: age <15 years, requiring medical attention due to a chronic health condition, diagnosed asthma, allergy toward peanuts, severe illness warranting hospital referral or emergency medical care, history of anaphylaxis or serious allergic reaction to any substance, pregnancy complications at the time of enrolment (moderate to severe oedema, blood Hb concentration <50 g/L, systolic blood pressure [BP] > 160 mmHg or diastolic BP > 100 mmHg), ever enrolled in the present trial during a previous pregnancy, or currently taking part in another clinical trial. The ethics committees of the University of Malawi College of Medicine, and the Pirkanmaa Hospital District, Finland approved the trial.

In both countries, haemoglobin concentration between 50 and 109 g/L (WHO, 2021) at baseline was not an exclusion criterion because the intervention provided at least daily iron and folic acid to all participants, and in addition, we monitored supplement intakes and morbidity bi‐weekly.

### 2.2 Baseline assessment and group assignments

We assessed women's baseline characteristics before randomisation. These included sociodemographic and household characteristics by questionnaire; weight (Seca 874), height (Seca 217) and blood haemoglobin concentration (Ghana: HemoCue AG; Malawi: HemoCue AB) by standard procedures; gestational age mainly by ultrasound biometry (Ghana: Aloka SSD 500; Malawi: EDAN DUS 3 Digital Ultrasonic Diagnostic Imaging System; EDAN Instruments Inc.); and malaria parasitemia by a rapid test (Ghana: Vision Biotech; Malawi: Clearview Malaria Combo, British Biocell International Ltd). We calculated a household assets index as a proxy indicator for socioeconomic status, and a household food insecurity access scale (HFIAS) score as a proxy indicator for food insecurity (Adu‐Afarwuah et al., 2015; Ashorn, Alho, Ashorn, Cheung, Dewey, Harjunmaa, et al., 2015).

After baseline assessments, pregnant women were randomised to one of three supplementation groups. In Ghana, women were assigned to receive daily: iron and folic acid capsule until childbirth and thereafter placebo (200 mg/day Ca) until the end of follow‐up at 6 months postpartum (IFA supplement or group), or multiple micronutrient capsule throughout follow‐up (MMN supplement or group); or 20 g/day SQ‐LNS throughout follow‐up (SQ‐LNS supplement or LNS group). In Malawi, women were assigned to receive one of the same three supplements as in Ghana until childbirth. After childbirth, only those women enrolled on the ‘complete follow‐up’ scheme (*n* = 864) received the assigned placebo or supplement and underwent a full follow‐up morbidity monitoring until 6 months postpartum (lactation) as in Ghana; those in the ‘simplified follow‐up’ scheme (*n* = 527) received no placebo or supplement postpartum and did not undergo any morbidity monitoring after childbirth.

The randomisation techniques in Ghana (Adu‐Afarwuah et al., 2015) and Malawi (Ashorn, Alho, Ashorn, Cheung, Dewey, Harjunmaa, et al., 2015) were similar: the Study Statistician at UC Davis used a computer‐generated (SAS version 9.4) scheme to develop the group assignments in blocks of 9, and each supplement or group was coded with three different colours (Adu‐Afarwuah et al., 2015) or alphabetical letters (Ashorn, Alho, Ashorn, Cheung, Dewey, Harjunmaa, et al., 2015). At each site, group assignments were placed in opaque envelopes and labelled in a manner inconspicuous to the women. Subsequently, the envelopes were stacked in increasing order of block numbers. At each enrolment, the individual performing the randomisation shuffled the nine (Adu‐Afarwuah et al., 2015) or six (Ashorn, Alho, Ashorn, Cheung, Dewey, Harjunmaa, et al., 2015) topmost envelopes in the stack, asked the potential participant to choose one (to determine the group assignment), and then returned the unused envelopes to the top of the stack. This process was repeated until all the envelopes for the enrolment site were used. When there were less than nine (Adu‐Afarwuah et al., 2015) or six (Ashorn, Alho, Ashorn, Cheung, Dewey, Harjunmaa, et al., 2015) envelopes left for participants to pick from, the individual performing the randomisation presented whatever number of envelopes that remained. It was not possible for anyone to guess the group assignments since none had knowledge of the randomisation scheme. Group allocation information was kept only by the field supervisor at the project site, and the Study Statistician at UC Davis.

### 2.3 Nutrient supplements

The nutrient contents of the supplements women consumed are shown in Table 1. The IFA capsule contained 60 mg/day of iron and 400 µg/day of folic acid reflecting the standard micronutrient supplementation for pregnant women in Ghana (WHO, 2012) and Malawi (Ashorn, Alho, Ashorn, Cheung, Dewey, Harjunmaa, et al., 2015) at the time of the trials. The MMN supplement contained 18 vitamins and minerals providing either *1x* or *2x* the recommended dietary allowance (RDA) of nutrients for pregnancy (Adu‐Afarwuah et al., 2015) except for iron, which we kept at 20 mg/day assuming that together with the amount from the usual diet, this dose would give a total daily intake close to the UNICEF/WHO/UNU International Multiple Micronutrient Preparation UNIMMAP iron content for pregnancy (UNICEF/WHO/UNU, 1999) without greatly exceeding the RDA (9 mg/day) for lactation (Adu‐Afarwuah et al., 2015; IOM, 2001). The SQ‐LNS contained the same micronutrients as the MMN group, and in addition, four minerals (calcium, phosphorus, potassium and magnesium), protein (2.6 g/day), energy (118 kcal/day), and EFAs (4.59 g/day linoleic acid and 0.59 g/day α‐linolenic acid). The oil content of the SQ‐LNS consisted of soybean oil and the oil supplied by the peanut paste ingredient.

**Table 1:** Composition of supplements used in the study.

Ration and nutrients	IFA^a^	MMN^b^	SQ‐LNS for women^c^, ^d^
Ration		1 tablet	20g/day
Total energy (kcal)		0	118
Protein (g)		0	2.6
Fat (g)		0	10
Linoleic acid (g)		0	4.59
α‐Linolenic acid (g)		0	0.59
Vitamin A (μg RE)		800	800
Vitamin C (mg)		100	100
Vitamin B_1_ (mg)		2.8	2.8
Vitamin B_2_ (mg)		2.8	2.8
Niacin (mg)		36	36
Folic acid (μg)	400	400	400
Pantothenic acid (mg)		7	7
Vitamin B_6_ (mg)		3.8	3.8
Vitamin B_12_ (μg)		5.2	5.2
Vitamin D (IU)		400	400
Vitamin E (mg)		20	20
Vitamin K (μg)		45	45
Iron (mg)	60	20	20
Zinc (mg)		30	30
Cu (mg)		4	4
Calcium (mg)		0	280
Phosphorus (mg)		0	190
Potassium (mg)		0	200
Magnesium (mg)		0	65
Selenium (μg)		130	130
Iodine (μg)		250	250
Manganese (mg)		2.6	2.6

Abbreviations: IFA, iron and folic acid capsule; MMN, multiple micronutrient supplement capsule; SQ‐LNS, small‐quantity lipid‐based nutrient supplement.

^a^ Reflected the standard micronutrient supplementation for pregnant women in Ghana (WHO, 2012) and Malawi (Ashorn, Alho, Ashorn, Cheung, Dewey, Harjunmaa, et al., 2015) at the time of the trials.

^b^ Micronutrient content was adapted from the UNICEF/WHO/UNU International Multiple Micronutrient Preparation UNIMMAP (UNICEF/WHO/UNU, 1999) formulation and a similar formulation used in Guinea Bissau (Kæstel et al., 2005), except iron.

^c^ Same micronutrient content as MMN.

^d^ Nutrient concentrations include contributions from the ingredients as well as from the multiple micronutrient premix. Oil content consisted of soybean oil and oil from the peanut paste ingredient, which were combined to meet the target amounts of α‐linolenic acid and linoleic acid in the final product.

The IFA and MMN (each in 10‐capsule blister packs) were produced by DSM South Africa, and the SQ‐LNSs (individual 20‐g sachets for women and 10‐g sachets for infants) by Nutriset S.A.S. (Malaunay, France).

### 2.4 Intervention

Intervention procedures in Ghana (Adu‐Afarwuah et al., 2015) and Malawi (Ashorn, Alho, Ashorn, Cheung, Dewey, Harjunmaa, et al., 2015) were similar. At enrolment, women received a 2‐week supply of the assigned supplement with the advice to those in the IFA and MMN groups to consume one capsule each day with water after a meal, and those in the LNS group to consume one sachet each day after mixing the entire content of the sachet with a small amount of food. In addition, women received the standard message: ‘Do not forget to eat meat, fish, eggs, fruits and vegetables whenever you can; you still need these foods even as you take the supplement we have given you’. We advised women not to consume more than one capsule (IFA and MMN groups) or sachet (LNS group) per day, even if they forgot to take the supplement the previous day or days and to take their assigned supplement along if they had to travel out of the study area. Women who travelled and did not return before the next biweekly visit were given an extra supply for the period they intended to be away.

From enrolment to childbirth (hereafter, pregnancy), field workers visited women in the homes biweekly during which they delivered fresh supplies of supplements or placebo and collected self‐reports of supplement intakes and morbidity. From childbirth till exit at 6 months postpartum (hereafter, lactation), field workers visited the women weekly (partly to collect infant morbidity data) but delivered the women's supplement or placebo and collected the women's morbidity data biweekly as before. To corroborate selfreports of supplement intake, field workers recovered and counted any unused supplements.

### 2.5 Morbidity symptoms data collection

At each biweekly visit in Ghana (Adu‐Afarwuah et al., 2015) and Malawi (Ashorn, Alho, Ashorn, Cheung, Dewey, Harjunmaa, et al., 2015), field workers asked and probed women about the number of days preceding the visit when women experienced morbidity symptoms. If a woman had a symptom during the days in question, she was asked whether the symptom was present ‘yesterday’ (yes/no). We considered 13 symptoms categorised into generalised (fever); gastrointestinal (poor appetite, nausea, vomiting, abdominal pain, diarrhoea and severe diarrhoea); reproductive (pregnancy‐related bleeding and mastitis); and respiratory (cough, sore throat, any nasal discharge and severe nasal discharge) morbidity. These symptoms were selected based on a similar study in Bangladesh (Kim et al., 2012) suggesting that they may be common in our study settings. Women were asked about abdominal pain and pregnancy‐related bleeding during pregnancy only, and mastitis during lactation only. We defined diarrhoea as the passage of ≥3 watery stools (Ghosh et al., 2010) or by women's report of increased frequency and reduced consistency of stools (Bhutta et al., 1991) in a 24‐h period, severe diarrhoea as having either bloody stools or mucus in stools or both and severe nasal discharge as having a thick or yellowish nasal discharge.

In Ghana, field workers unable to complete the morbidity data collection on the usual 14‐day schedule (typically because of a participant's travel) had up to 7 days to complete the visit or else consider it missed. At the first home visit 14–21 days after enrolment, field workers collected data ‘since enrolment’; in subsequent visits, data were collected ‘since last visit’ if the current visit occurred within 7–21 days after the preceding visit, or ‘during the past 2 weeks’ if the current visit occurred >21 days after the preceding visit. With this data collection scheme, it was possible to obtain self‐reports of women's morbidity over all or nearly all of the entire follow‐up period.

In Malawi, the first home visit was completed within 7–13 days after enrolment, the second occurred 7 or 14 days after the first visit, and subsequent visits were completed every 14 days thereafter. At Weeks 32 and 36 of pregnancy, supplement delivery and data collection were accomplished during the women's visits to the laboratory. Although women were visited biweekly, the recall of self‐reported morbidity was conducted over the ‘past 7 days’ because of fears that recall over a longer period might be problematic due to a low average level of education.

It was not possible to blind field workers or study participants to those who received the SQ‐LNSs, because the capsules (IFA and MMN) and SQ‐LNS sachets looked different. As we described previously (Adu‐Afarwuah et al., 2016; Ashorn, Alho, Ashorn, Cheung, Dewey, Gondwe, et al., 2015; Klevor et al., 2016), self‐reported adherence to supplement intake (percentage of days when the supplement was reportedly consumed) during pregnancy/lactation was 88%/86% for the IFA group, 87%/85% for the MMN group, and 84%/80% for the LNS group in Ghana, and 92%/97% for the IFA group, 91%/97% for the MMN group, and 94%/96% for the LNS group in Malawi (Klevor et al., 2016).

### 2.6 Measurement time intervals and outcome measures

Because the appearance of certain morbidity symptoms is related to the duration of the pregnancy (e.g., nausea and vomiting often appear and peak during the first trimester, and disappear by the end of the second trimester (Lacasse & Bérard, 2008), we defined three measurement intervals during pregnancy, namely: (i) the first trimester (0–90 days), (ii) second trimester (91–180 days) and (iii) third trimester (181 days) (Taylor et al., 2017). For lactation, we defined two intervals, each with a 3‐month duration as done for pregnancy: (i) 0–90 days after delivery and (ii) 91–180 days after delivery.

For each measurement interval, we created two variables for each morbidity symptom. First, to calculate the period prevalence of each symptom, we created a binary variable with a value of 1 (if the woman reported the symptom for ≥1 day during the period in question) or 0 (if the woman never reported the symptom during the period in question). For example, for diarrhoea during the second trimester of pregnancy, the binary variable created had a value of 1 if the sum of the days the woman reported diarrhoea during the period was ≥1, and 0 if the sum was 0.

Second, we calculated the percentage of days with each symptom during the period in question, based on the number of days of monitoring for each woman. For example, for diarrhoea during the second trimester of pregnancy, we summed the number of days the woman reported diarrhoea during the period, divided the sum by the number of days for which morbidity was monitored for the woman during that period, and multiplied the result by 100.

### 2.7 Sample size and statistical analysis

The sample sizes in the Ghana and Malawi trials were based on detecting a small‐to‐moderate Cohen's effect size *d* of 0.3 between any two groups for either of the two primary continuous variables (i.e., length‐for‐age z‐score at birth and at 18 months of age), with a two‐sided 5% test and 80% power. As described previously, the number of pregnant women enrolled in Ghana was 1320 (Adu‐Afarwuah et al., 2015). Due to the temporary mislabeling of IFA and MMN capsules, which we described previously (Adu‐Afarwuah et al., 2015), 170 women initially assigned to the IFA group inadvertently received MMN either throughout (*n* = 85) or during part of (*n* = 85) pregnancy before receiving the intended IFA supplement. Likewise, 170 women initially assigned to MMN inadvertently received IFA either throughout (*n* = 78) or during part of (*n* = 92) pregnancy before receiving the intended MMN supplement. We elected to include all the enrolled women in the current analysis and to perform the analysis by the groups to which women were assigned at enrolment, because the percentage of follow‐up days during which the women in the IFA and MMN groups had the unintended exposure was relatively small (13%) (Adu‐Afarwuah et al., 2016), and no women in the LNS group received any other supplement apart from the SQ‐LNSs.

In Malawi, a total of 1391 women were enrolled (Ashorn, Alho, Ashorn, Cheung, Dewey, Harjunmaa, et al., 2015), but those in the ‘simplified follow‐up’ scheme (*n* = 527) were not included in the morbidity analysis postpartum because those women received no placebo or supplement and did not undergo any morbidity monitoring postpartum.

Our statistical analysis plan was posted online (https://ilins.ucdavis.edu/) before analysis. We used SAS version 9.4 (SAS Institute) to perform the analyses according to the complete‐case intention‐to‐treat principle (Groenwold et al., 2014) and the group to which women were assigned, regardless of any protocol violations. Data on women who were lost to follow‐up for any reason were included in the analysis if available. Background and household variables were summarised using percentages for categorical variables and mean ± SD for continuous variables. We examined morbidity variables by time interval, that is, the second and third trimesters during pregnancy and 0–3 and 3–6 months during lactation, after excluding the first trimester due to a small number of women who attended antenatal clinics and were enrolled during the first trimester. We evaluated the effects of time interval and intervention group (IFA, MMN or LNS) on the occurrence of morbidity symptoms (yes/no) by using repeated measures logistic regression, and on the percentage of days with morbidity symptoms by using repeated measures analysis of variance (ANOVA). The fixed effects in these models were one within‐subjects factor (i.e., time intervals during pregnancy and lactation) and one between‐subjects factor (intervention group), while treating participants as a random effect. We used a two‐tailed test for the analysis of all outcome variables because we did not predict the direction of any group differences. We set the level of significance (α) at 0.05 and considered 0.05 < *p* ≤ 0.08 to indicate a tendency toward significance (Curran‐Everett & Benos, 2004; Pintó et al., 2019).

The analyses were conducted in two stages. In the first stage, we fitted a logistic regression or ANOVA model that included time interval, intervention group, and their interaction (time interval × intervention group). When there was a significant intervention effect (*p* < 0.05) or intervention × time interval interaction (*p* < 0.1), we evaluated the intervention effect for each time interval separately (i.e., cross‐sectionally) in the second stage of the analysis to identify the time interval or intervals during which any group differences occurred. When the intervention effect did not differ by time interval (*p*‐for‐interaction ≥ 0.1) we removed the interaction term from the model in the second stage of the analysis, so that inference was based on the intervention effect over the entire follow‐up period.

In adjusted analysis, we controlled for prespecified baseline variables, including age, height, BMI, GA, years of education, marital status, household assets index (proxy indicator for socioeconomic status), HFIAS score, the season in which woman was enrolled, primiparity, blood haemoglobin concentration, and positive malaria RDT if they were significantly associated with the outcome at 10% level of significance. Finally, we performed two additional exploratory analyses: first, we used a ‘per protocol’ sensitivity analysis to assess the treatment effect among women considered to have high adherence to supplementation (i.e., added supplements to their foods >70% of days during follow‐up); second, we evaluated potential effect modification (*p*‐for‐interaction) by seven baseline characteristics, including age, GA, years of education, household assets index, household toilet facility category, HFIAS score, and primiparity. We did not perform stratified analyses because the effect modification analyses were exploratory.

## 3 RESULTS

The background characteristics of the women enrolled in the iLiNS DYAD trials are shown in Table 2. In Ghana, the women's age averaged ~27 years, formal education averaged 7 completed years, and mean gestational age and BMI at enrolment were 16.1 weeks and 24.8 kg/m^2^, respectively (with a prevalence of overweight or obesity (BMI ≥ 25 kg/m^2^) being about 41%). The average household assets index and HFIAS score suggest moderate socioeconomic status and low household food insecurity. About 34% of the women were first‐time mothers, 51% were enrolled in the dry season, and 10% tested positive for malaria.

**Table 2:** Background characteristics of women enrolled in the iLiNS DYAD trials in Ghana and Malawi, by group as randomised.^a^

Background characteristics	Ghana			Malawi		
	IFA (*n* = 441)	MMN (*n* = 439)	LNS (*n* = 440)	IFA (*n* = 463)	MMN (*n* = 466)	LNS (*n* = 462)
Age (years)	26.4 ± 5.6 (441)	26.9 ± 5.4 (439)	26.9 ± 5.6 (440)	25.0 ± 6.1 (463)	24.8 ± 6.1 (466)	25.1 ± 6.2 (462)
Formal education (years)	7.8 ± 3.5 (441)	7.6 ± 3.6 (439)	7.6 ± 3.9 (440)	3.9 ± 3.4 (448)	4.1 ± 3.4 (437)	4.1 ± 3.6 (441)
Gestational age at enrolment (weeks)	16.0 ± 3.3 (440)	16.2 ± 3.2 (436)	16.1 ± 3.3 (435)	16.8 ± 2.1 (463)	16.8 ± 2.1 (466)	16.9 ± 2.2 (462)
Body mass index (kg/m^2^)	24.6 ± 4.5 (433)	24.7 ± 4.4 (432)	25.0 ± 4.6 (435)	22.2 ± 2.7 (460)	22.1 ± 2.7 (462)	22.2 ± 3.1 (460)
Overweight/obesity, *n*/total *n* (%)	173/433 (40.0)	169/432 (39.1)	187/435 (43.0)	68/460 (14.8)	52/462 (11.3)	66/460 (14.3)
Assets index^b^	0.04 ± 1.03 (433)	0.06 ± 0.97 (431)	−0.09 ± 1.00 (432)	−0.02 ± 1.00 (443)	−0.01 ± 0.97 (431)	0.02 ± 1.03 (439)
HFIAS score^c^	2.6 ± 4.4 (434)	2.7 ± 4.3 (431)	2.6 ± 4.0 (432)	5.0 ± 4.5 (447)	5.2 ± 4.7 (434)	4.6 ± 4.2 (440)
Primiparous women, *n*/total *n* (%)	156/441 (35.4)	143/439 (32.6)	147/440 (33.4)	94/462 (20.3)	107/465 (23.0)	102/461 (22.1)
Season at enrolment = dry season	226/441 (51.2)	224/439 (51.0)	225/440 (51.1)	253/463 (54.6)	266/466 (57.1)	265/462 (57.4)
Tested positive for malaria, *n*/total *n* (%)^d^	37/440 (8.4)	42/439 (9.6)	54/440 (12.3)	105/463 (22.7)	112/465 (24.1)	105/460 (22.8)

Abbreviations: IFA, iron and folic acid capsule; MMN, multiple micronutrient supplement capsule; LNS, lipid‐based nutrient supplement.

^a^ The iLiNS‐DYAD trials were International Lipid‐based Nutrient Supplements trials in which mother‐child dyads were enrolled.

^b^ Assets index (a composite of household ownership of assets such as radio, television, refrigerator, cell phone and stove) serves as a proxy indicator for household socioeconomic status (SES). Higher values are indicative of higher SES.

^c^ Household Food Insecurity Access Scale (HFIAS) score is a continuous measure of the degree of food insecurity based on a set of questions that encompass three domains of food insecurity: (i) anxiety and uncertainty about the household food supply, (ii) insufficient quality and (iii) insufficient food intake and its physical consequences (Coates et al., 2007). Higher values are indicative of higher household food insecurity.

^d^ Rapid Diagnostic Test (Clearview Malarial Combo, Vision Biotech) detected *Plasmodium. falciparum* and non‐*Plasmodium falciparum* histidine‐rich protein‐2.

Among the women enrolled in Malawi, the average age was ~25 years, the average completed years of formal education was 4, and the mean gestational age and BMI at enrolment were 16.8 weeks and 22.2 kg/m^2^, respectively (with the prevalence of overweight or obesity being 13.5%). The average household assets index and HFIAS score suggest that the women's socioeconomic status was mostly low, and their household food insecurity was generally high. About 21.8% of the women were first‐time mothers, 56% were enrolled in the dry season, and 23% tested positive for malaria.

During the second and third trimesters of pregnancy, morbidity data were available for up to 1243 women in Ghana and up to 1200 women in Malawi. Postpartum, morbidity data were available for up to 1212 women in Ghana and up to 730 women in Malawi, given that women in the ‘simplified follow‐up’ scheme in Malawi did not undergo any morbidity monitoring postpartum. The mean ± SD number of days of morbidity monitoring in the second trimester, third trimester, 0‐3 mo lactation and 3–6 months lactation were 56 ± 19, 89 ± 18, 81 ± 13 and 80 ± 12, respectively for Ghana, and 29 ± 9, 40 ± 10, 36 ± 9 and 38 ± 9, respectively for Malawi. In both countries, the mean number of days of morbidity monitoring was smallest for the second trimester compared with the other periods, since many of the women were enrolled during the second trimester.

### 3.1 Summary of morbidity results by time interval

Table 3 summarises the prevalence (percentage of women with) and mean ± SD percentage of days women had morbidity symptoms during pregnancy and lactation, by time interval, in Ghana and Malawi. In Ghana, abdominal pain, vomiting and nausea were the most common morbidity symptoms during the second trimester of pregnancy (44.6%–53.6% period prevalence; 5.3–8.6 mean percentage of days), while abdominal pain, nasal discharge and cough were the most common symptoms during the third trimester (42.1%–56.8% period prevalence; 4.0–10.1 mean percentage of days). During lactation, nasal discharge, cough and fever were the most common symptoms during both the 0–3 months period (17.2%–24.5% period prevalence; 1.2 mean percentage of days) and the 3–6 months period (17.9%–26.6% period prevalence; 1.2–1.3 mean percentage of days).

**Table 3:** Summary of prevalence and percentage of days of monitoring when women had morbidity symptoms in Ghana and Malawi, by time interval during pregnancy and lactation.^a^

	Ghana (*n* = 1320)^b^			Malawi (*n* = 1391)^b^		
	Prevalence		Percentage of monitoring days	Prevalence		Percentage of monitoring days
Morbidity and time interval	*n*/total *n*	%	$\bar{x}\;\pm$ SD (*n*)	*n*/total *n*	%	$\bar{x}\;\pm$ SD (*n*)
Fever						
Second trimester	454/1243	36.5	6.3 ± 12.4 (1194)	395/1118	35.3	6.3 ± 12.4 (1118)
Third trimester	465/1243	37.4	5.3 ± 10.0 (1182)	436/1200	36.3	5.3 ± 10.0 (1200)
0–3 months lactation	208/1212	17.2	2.0 ± 6.1 (1191)	128/730	17.5	2.0 ± 6.1 (730)
3–6 months lactation	243/1179	20.6	1.8 ± 5.9 (1138)	110/719	15.3	1.8 ± 5.9 (719)
Poor appetite						
Second trimester	408/1243	32.8	3.8 ± 8.7 (1191)	384/1118	34.4	5.8 ± 11.6 (1118)
Third trimester	433/1243	34.8	3.0 ± 7.1 (1176)	388/1200	32.3	4.0 ± 8.7 (1200)
0–3 months lactation	162/1212	13.4	1.0 ± 4.3 (1184)	52/730	7.1	0.8 ± 3.7 (730)
3–6 months lactation	150/1179	12.7	0.8 ± 3.1 (1154)	53/720	7.4	0.9 ± 4.6 (719)
Nausea						
Second trimester	554/1243	44.6	7.3 ± 14.7 (1199)	419/1118	37.5	8.2 ± 16.2 (1118)
Third trimester	430/1243	34.6	3.9 ± 10.3 (1205)	398/1200	33.2	5.2 ± 11.4 (1200)
0–3 months lactation	118/1212	9.7	0.6 ± 3.5 9 (1199)	77/730	10.6	1.3 ± 5.3 (730)
3–6 months lactation	128/1179	10.9	0.8 ± 3.6 (1167)	64/720	8.9	1.2 ± 5.8 (720)
Vomiting						
Second trimester	557/1243	44.8	5.3 ± 11.8 (1229)	267/1118	23.8	3.5 ± 9.8 (1118)
Third trimester	462/1243	37.2	2.7 ± 7.6 (1224)	251/1200	20.9	2.4 ± 7.3 (1200)
0–3 months lactation	63/1212	5.2	0.1 ± 0.7 (1211)	42/730	5.8	0.4 ± 2.5 (730)
3–6 months lactation	58/1179	4.9	0.1 ± 1.3 (1176)	37/720	5.1	0.6 ± 3.8 (720)
Abdominal pain						
Second trimester	626/1167	53.6	8.6 ± 15.9 (1121)			
Third trimester	704/1239	56.8	10.1 ± 17.2 (1188)			
Severe diarrhoea						
Second trimester	89/1243	7.2	0.4 ± 2.4 (1243)	101/1118	9.0	1.0 ± 4.0 (1118)
Third trimester	88/1243	7.1	0.3 ± 1.9 (1241)	131/1200	10.9	0.9 ± 3.6 (1200)
0–3 months lactation	24/1212	2.0	0.1 ± 0.8 (1211)	40/730	5.5	0.6 ± 2.9 (730)
3–6 months lactation	20/1179	1.7	0.1 ± 0.8 (1179)	37/720	5.1	0.6 ± 4.5 (720)
Mastitis						
0–3 months lactation	83/1212	6.9	0.5 ± 3.1 (1191)	35/730	4.8	0.8 ± 4.1 (730)
Cough						
Second trimester	464/1243	37.3	4.6 ± 10.3 (1170)	550/1118	49.2	12.3 ± 17.7 (1118)
Third trimester	523/1243	42.1	4.0 ± 8.7 (1156)	667/1200	55.6	11.2 ± 14.9 (1200)
0–3 months lactation	225/1212	18.6	1.2 ± 4.8 (1160)	229/730	31.4	5.5 ± 10.6 (730)
3–6 months lactation	211/1179	17.9	1.3 ± 4.5 (1129)	209/720	29.0	4.8 ± 10.7 (720)
Sore throat						
Second trimester	145/1243	11.7	0.9 ± 4.1 (1213)	344/1118	30.8	6.2 ± 12.5 (1118)
Third trimester	178/1243	14.3	0.8 ± 3.7 (1210)	421/1200	35.1	5.9 ± 10.8 (1200)
0–3 months lactation	57/1212	4.7	0.2 ± 1.0 (1198)	119/730	16.3	2.3 ± 6.7 (730)
3–6 months lactation	75/1179	6.4	0.3 ± 1.4 (1166)	128/720	17.8	2.5 ± 8.1 (720)
Nasal discharge						
Second trimester	515/1243	41.4		354/1118	31.7	7.2 ± 14.4 (1118)
Third trimester	539/1243	43.4		394/1200	32.8	5.6 ± 10.7 (1200)
0–3 months lactation	297/1212	24.5		140/730	19.2	2.8 ± 7.6 (730)
3–6 months lactation	313/1179	26.6		129/720	17.9	2.7 ± 8.4 (720)
Severe nasal discharge						
Second trimester	291/1243	23.4	2.6 ± 7.9 (1178)	142/1118	12.7	2.6 ± 8.5 (1118)
Third trimester	334/1243	26.9	1.9 ± 5.6 (1174)	160/1200	13.3	1.8 ± 5.7 (1200)
0–3 months lactation	184/1212	15.2	0.8 ± 3.4 (1169)	49/730	6.7	0.9 ± 4.0 (730)
3–6 months lactation	199/1179	16.9	1.1 ± 4.2 (1131)	46/720	6.4	1.0 ± 5.6 (720)

^a^ Ghana: All enrolled women received intervention or placebo and underwent a full follow‐up morbidity monitoring until 6 months postpartum. Malawi: All enrolled women received intervention and underwent a full follow‐up morbidity monitoring until childbirth; thereafter, only women (*n* = 864) in the ‘complete follow‐up’ scheme received the assigned placebo or intervention and underwent a full follow‐up morbidity monitoring until 6 months postpartum. The mean ± SD number of days of morbidity monitoring in the second trimester, third trimester, 0‐3 months lactation and 3–6 months lactation were 56 ± 19, 89 ± 18, 81 ± 13 and 80 ± 12, respectively for Ghana and 29 ± 9, 40 ± 10, 36 ± 9 and 38 ± 9, respectively for Malawi.

^b^ *p*‐values for these analyses were not reported because the study did not have prespecified hypotheses for the effect of time interval on the prevalence or percentage of days of monitoring when women had morbidity symptoms.

In Malawi, cough, nausea and fever were the most common morbidity symptoms during the second trimester of pregnancy (35.3%–49.1% period prevalence; 6.3–12.3 mean percentage of days), while cough, fever and sore throat were the most common symptoms during the third trimester (35.1%–55.7% period prevalence; 5.3–11.2 mean percentage of days). During lactation, cough, nasal discharge and fever were the most common symptoms during the 0–3 months period (17.5%–31.4% period prevalence; 2.0–5.5 mean percentage of days), while cough, nasal discharge and sore throat were the most common during the 3–6 months period (17.8%–29.0% period prevalence; 2.5–4.8 mean percentage of days).

In both countries, the main effect of time interval was statistically significant for all morbidity outcomes, except for the prevalence of abdominal pain, which was assessed during pregnancy (only) in Ghana but not during any time interval in Malawi. For most gastrointestinal symptoms (notably poor appetite, nausea and vomiting), the prevalence or mean percentage of days declined significantly from the second trimester of pregnancy through 0–3 months or 3–6 months of lactation. A similar pattern was shown for the mean percentage of days of monitoring when women had fever and respiratory symptoms (cough, sore throat, any nasal discharge and severe nasal discharge), but the prevalence of fever and respiratory symptoms increased or appeared to increase from the second to the third trimesters of pregnancy, before declining through 0–3 months or 3–6 months of lactation.

### 3.2 Main effect of the intervention group

Table 4 shows the prevalence of morbidity symptoms during pregnancy and lactation, by intervention group, in Ghana and Malawi. In Ghana, the three intervention groups did not differ significantly in the prevalence of the morbidity symptoms during the follow‐up period, except for vomiting (*p* = 0.046). Analyses of the separate time intervals showed that the group differences in vomiting prevalence occurred only during the third trimester of pregnancy (*p* = 0.019) and at 0–3 months lactation (*p* = 0.025). In the third trimester of pregnancy, vomiting prevalence was greater in the MMN group (41.4%) than the LNS group (32.0%) and the odds ratio (95% confidence interval [CI]) of vomiting in the MMN group compared with the LNS group was 1.50 (1.13, 2.00); the IFA group (37.9%) did not differ significantly from the other groups. At 0–3 months of lactation, vomiting prevalence was greater in the MMN group (7.5%) than in the IFA group (3.2%) and the odds ratio (95% CI) of vomiting in the MMN compared with the IFA group was 2.48 (1.28, 4.83); the LNS group (5.0%) did not differ significantly from the other two groups.

**Table 4:** Prevalence of morbidity symptoms during pregnancy and lactation, by intervention group, in Ghana and Malawi^a^.

	Ghana^b^								Malawi^c^							
	IFA		MMN		LNS		*p* d	*p*‐int	IFA		MMN		LNS		*P* d	*P*‐int
	*n*/total *n*	%	*n*/total *n*	%	*n*/total *n*	%			*n*/total *n*	%	*n*/total *n*	%	*n*/total *n*	%		
Fever																
Overall	449/1650	27.2	458/1623	28.3	462/1604	28.8	0.71	0.66	334/1254	26.6	379/1266	29.9	357/1247	28.6	0.28	0.97
Poor appetite																
Overall	393/1650	23.8	399/1623	24.6	361/1604	22.5	0.50	0.91	306/1255	24.4	289/1266	22.9	281/1247	22.5	0.56	0.34
Nausea																
Overall	384/1650	23.3	422/1623	26.0	423/1604	26.4	0.16	0.13	314/1255	25.0	339/1266	26.8	306/1247	24.5	0.47	0.89
Vomiting																
Overall	382/1650	23.2^a,b^	415/1623	25.6^a^	343/1604	21.4^b^	0.046	0.19	200/1255	15.9	211/1266	16.7	186/1247	14.9	0.53	0.06
Second trimester	191/417	45.8	192/418	45.9	174/408	42.6	0.56		95/372	25.5	98/378	25.9	73/368	19.8	0.10	
Third trimester	159/419	37.9^a,b^	172/415	41.4^a^	131/409	32.0^b^	0.019		71/397	17.9	89/402	22.1	91/401	22.7	0.19	
0‐3 mo lactation	13/412	3.2^a^	30/401	7.5^b^	20/399	5.0^a,b^	0.025		16/246	6.5	15/242	6.2	11/242	4.5	0.61	
3‐6 mo lactation	19/402	4.7	21/389	5.4	18/388	4.6	0.87		17/240	7.1	9/244	3.7	11/236	4.7	0.23	
Abdominal pain																
Overall	454/807	56.3	449/805	55.8	427/794	53.8	0.66	0.43								
Severe diarrhoea																
Overall	81/1650	4.9	71/1623	4.4	69/1604	4.3	0.68	0.44	105/1255	8.4	103/1266	8.1	101/1247	8.1	0.95	0.05
Second trimester									40/372	10.8	32/378	8.5	29/368	7.9	0.36	
Third trimester									40/397	10.1	47/402	11.7	44/401	11.0	0.76	
0‐3 mo lactation									14/246	5.7	17/242	7.0	9/242	3.7	0.28	
3‐6 mo lactation									11/240	4.6^a,b^	7/244	2.9^b^	19/236	8.1^a^	0.041	
Mastitis																
Overall	30/814	3.7	30/790	3.8	33/787	4.2	0.86	0.40	23/486	4.7	16/486	3.3	13/478	2.7	0.30	0.95
Cough																
Overall	490/1650	29.7	459/1623	28.3	475/1604	29.6	0.64	0.30	526/1255	41.9	573/1255	45.3	555/1247	44.5	0.30	0.31
Sore throat																
Overall	158/1650	9.6	157/1623	9.7	140/1604	8.7	0.58	0.99	343/1255	27.3	341/1255	26.9	328/1247	26.3	0.87	0.76
Nasal discharge																
Overall	579/1650	35.1	524/1623	32.3	560/1604	34.9	0.26	0.67	334/1255	26.6	348/1255	27.5	335/1247	26.9	0.91	0.41
Severe nasal discharge																
Overall	351/1650	21.3	316/1623	19.5	340/1604	21.2	0.49	0.45	126/1255	10.0	139/1255	11.0	133/1247	10.7	0.75	0.24

*Note*: *p*‐int indicates whether intervention effect differed by time interval. When there was a significant intervention effect (*p* < 0.05) or intervention × time interval interaction (*p* < 0.1), we evaluated the impact of intervention group for each time interval separately to identify the time interval or intervals during which any group differences occurred. Abbreviations: IFA, iron and folic acid capsule; MMN, multiple micronutrient supplement capsule; LNS, lipid‐based nutrient supplement.

^a^ The mean (SD) number of days of morbidity monitoring in the second trimester, third trimester, 0–3 months lactation and 3–6 months lactation were 56 ± 19, 89 ± 18, 81 ± 13 and 80 ± 12, respectively for Ghana and 29 ± 9, 40 ± 10, 36 ± 9 and 38 ± 9, respectively for Malawi.

^b^ Total *n* = 1320 enrolled in Ghana, by group: IFA (*n* = 441), MMN (*n* = 439), LNS (*n* = 440). All enrolled women received intervention or placebo and underwent a full follow‐up morbidity monitoring until 6 months postpartum.

^c^ Total *n* = 1391 enrolled in Malawi, by group: IFA (*n* = 463), MMN (*n* = 466), LNS (*n* = 462). All enrolled women received intervention and underwent a full follow‐up morbidity monitoring until childbirth; thereafter, only women (*n* = 864) in the ‘complete follow‐up’ scheme received the assigned placebo or intervention and underwent a full follow‐up morbidity monitoring until 6 months postpartum. Prevalence of abdominal pain was not measured in Malawi.

^d^ *p*‐value indicates the impact of intervention group on the prevalence of morbidity symptoms at *α* = 0.05, by repeated measures logistic regression.

In Malawi, the period prevalence of morbidity symptoms during the follow‐up period did not differ significantly among the three groups. We found a significant intervention × time interval interaction for vomiting and severe diarrhoea. Within the separate time intervals, the groups differed only in the prevalence of severe diarrhoea during 3–6 months lactation (*p* = 0.041), when prevalence was greater in the LNS group (8.1%) than the MMN group (2.9%) and the odds ratio (95% CI) of severe diarrhoea in the LNS group compared with the MMN group was 2.96 (1.22, 7.19); the IFA group (4.6%) did not differ significantly from the other groups. Adjusting for relevant baseline characteristics (results not shown) did not alter the findings in either country.

The mean ± SD percentage of days of monitoring when women had morbidity symptoms during pregnancy and lactation, by intervention group, in Ghana and Malawi are shown in Table 5. In Ghana, we found significant group differences (*p* = 0.002) and intervention × time interval interaction (*p* = 0.03) for mean ± SD percentage of days women had nausea during the follow‐up period. In the analysis based on the separate time intervals, the differences in the mean percentage of days women had nausea occurred during the third trimester of pregnancy (*p* = 0.040) and 0–3 months of lactation (*p* = 0.030). In the third trimester of pregnancy, the mean percentage of days women had nausea was greater in the LNS (4.6 ± 12.1) group than in the IFA group (2.8 ± 6.8) and the difference in mean (95% CI) was 1.8 (0.4, 3.2); the MMN (4.8 ± 11.3) did not differ significantly from the other groups. At 0–3 months of lactation, the mean percentage of days women had nausea was greater in the MMN (0.8 ± 4.0) and LNS (0.7 ± 4.2) groups than in the IFA group (0.3 ± 1.7); the difference in mean (95% CI) when comparing the MMN and LNS groups with the IFA group was 0.5 (0.0, 1.0) for the MMN group and 0.4 (0.0, 0.9) for the LNS group. There were significant intervention × time interval interactions for the percentage of days of monitoring when women had mastitis (*p* = 0.04) and severe nasal discharge (*p* = 0.05), but in none of the separate time intervals did the groups differ in either of these outcomes.

**Table 5:** Mean ± SD percentage of monitoring days when women had morbidity symptoms during pregnancy and lactation, by intervention group, in Ghana and Malawi^a^.

	Ghana^b^					Malawi^c^				
	IFA $\bar{x}$ ± SD (*n*)	MMN $\bar{x}$ ± SD (*n*)	LNS $\bar{x}$ ± SD (*n*)	*p* d	*p*‐int	IFA $\bar{x}$ ± SD (*n*)	MMN $\bar{x}$ ± SD (*n*)	LNS $\bar{x}$ ± SD (*n*)	*p* d	*p*‐int
Fever										
Overall	2.2 ± 6.1 (1587)	2.6 ± 7.4 (1562)	2.6 ± 7.4 (1556)	0.46	0.36	3.9 ± 9.0 (1254)	4.6 ± 10.2 (1266)	4.4 ± 10.0 (1247)	0.30	0.82
Poor appetite										
Overall	2.1 ± 6.3 (1588)	2.3 ± 6.4 (1565)	2.2 ± 6.3 (1552)	0.92	0.74	3.5 ± 9.4 (1255)	3.3 ± 8.4 (1266)	3.2 ± 8.1 (1247)	0.52	0.85
Nausea										
Overall	2.7^a^ ± 8.4 (1613)	3.3^b^ ± 10.4 (1585)	3.5^b^ ± 10.3 (1572)	0.002	0.03	4.5 ± 12.1 (1255)	4.8 ± 11.8 (1266)	4.3 ± 11.4 (1247)	0.40	0.54
Second trimester	7.3 ± 14.0 (400)	7.3 ± 15.6 (404)	7.4 ± 14.5 (395)	0.99						
Third trimester	2.8^a^ ± 6.8 (408)	4.2^b^ ± 11.3 (401)	4.6^b^ ± 12.1 (396)	0.040						
0–3 months lactation	0.3^a^ ± 1.7 (407)	0.8^b^ ± 4.0 (396)	0.7^b^ ± 4.2 (396)	0.030						
3–6 months lactation	0.5 ± 2.6 (398)	0.8 ± 3.5 (384)	1.0 ± 4.5 (385)	0.15						
Vomiting										
Overall	2.0 ± 6.7 (1639)	2.2 ± 7.5 (1610)	2.0 ± 7.9 (1591)	0.59	0.10	1.9 ± 6.8 (1255)	2.2 ± 7.6 (1266)	1.9 ± 7.0 (1247)	0.80	0.15
Abdominal pain										
Overall	9.9 ± 17.2 (775)	9.0 ± 15.8 (769)	9.4 ± 16.7 (765)	0.66	0.44					
Severe diarrhoea										
Overall	0.2 ± 1.4 (1650)	0.2 ± 2.0 (1621)	0.2 ± 1.6 (1603)	0.94	0.49	0.9 ± 4.2 (1255)	0.8 ± 3.8 (1266)	0.7 ± 3.3 (1247)	0.66	0.20
Mastitis										
Overall	0.3 ± 2.5 (806)	0.4 ± 2.9 (786)	0.2 ± 1.7 (777)	0.13	0.04	0.9 ± 6.1 (486)	0.4 ± 2.9 (486)	0.5 ± 3.3 (478)	0.07	0.39
0–3 months lactation	0.5 ± 3.6 (404)	0.6 ± 3.1 (398)	0.4 ± 2.3 (389)	0.80						
Cough										
Overall	2.9 ± 8.2 (1557)	2.8 ± 7.7 (1535)	2.7 ± 7.0 (1523)	0.70	0.11	8.9 ± 14.7 (1255)	10 ± 15.5 (1266)	8.7 ± 14.1 (1247)	0.09	0.38
Sore throat										
Overall	0.5 ± 2.9 (1611)	0.6 ± 3.2 (1594)	0.5 ± 2.6 (1582)	0.83	0.91	4.6 ± 10.2 (1255)	4.9 ± 10.8 (1266)	4.4 ± 10.0 (1247)	0.71	0.81
Nasal discharge										
Overall						4.8 ± 11.1 (1255)	5.5 ± 12.0 (1266)	4.6 ± 10.4 (1247)	0.23	0.98
Severe nasal discharge										
Overall	1.5 ± 5.1 (1571)	1.6 ± 6.2 (1551)	1.6 ± 5.5 (1530)	0.79	0.05	1.5 ± 6.2 (1255)	2.1 ± 7.6 (1266)	1.5 ± 5.3 (1247)	0.39	0.06
Second trimester	2.6 ± 7.2 (391)	3.0 ± 9.5 (400)	2.2 ± 6.7 (387)	0.48		2.2 ± 7.5 (372)	3.8 ± 10.9 (378)	1.8 ± 6.3 (368)	0.07	
Third trimester	1.5 ± 4.3 (400)	1.8 ± 5.7 (390)	2.3 ± 6.5 (384)	0.20		1.5 ± 4.9 (397)	2.0 ± 6.7 (402)	1.9 ± 5.4 (401)	0.68	
0–3 months lactation	0.8 ± 3.9 (393)	0.7 ± 3.3 (387)	0.9 ± 3.0 (389)	0.75		0.7 ± 3.2 (246)	0.7 ± 3.3 (242)	1.2 ± 5.3 (242)	0.51	
3–6 months lactation	1.2 ± 3.9 (387)	0.9 ± 3.9 (374)	1.1 ± 4.7 (370)	0.69		1.2 ± 7.8 (240)	1.0 ± 5.1 (244)	0.7 ± 3.0 (236)	0.71	

*Note*: *p*‐int indicates whether intervention effect differed by time interval. When there was a significant intervention effect (*p* < 0.05) or intervention × time interval interaction (*p* < 0.1), we evaluated the impact of intervention group for each time interval separately to identify the time interval or intervals during which any group differences occurred. Abbreviations: IFA, iron and folic acid capsule; MMN, multiple micronutrient supplement capsule; LNS, lipid‐based nutrient supplement.

^a^ The mean ± SD number of days of morbidity monitoring in the second trimester, third trimester, 0–3 months lactation and 3–6 months lactation were 56 ± 19, 89 ± 18, 81 ± 13 and 80 ± 12, respectively for Ghana and 29 ± 9, 40 ± 10, 36 ± 9 and 38 ± 9, respectively for Malawi.

^b^ Total *n* = 1320 enrolled in Ghana, by group: IFA (*n* = 441), MMN (*n* = 439), LNS (*n* = 440). All enrolled women received intervention or placebo and underwent a full follow‐up morbidity monitoring until 6 months postpartum. Percentage of monitoring days with nasal discharge was not measured in Ghana.

^c^ Total *n* = 1391 enrolled in Malawi, by group: IFA (*n* = 463), MMN (*n* = 466), LNS (*n* = 462). All enrolled women received intervention and underwent a full follow‐up morbidity monitoring until childbirth; thereafter, only women (*n* = 864) in the ‘complete follow‐up’ scheme received the assigned placebo or intervention and underwent a full follow‐up morbidity monitoring until 6 months postpartum. Percentage of monitoring days with abdominal pain was not measured in Malawi.

^d^ *p*‐value indicates the impact of intervention group on the mean percentage of days of monitoring when women had morbidity symptoms within country, by repeated measures analysis of variance (ANOVA). For nausea, mastitis and severe nasal discharge, the impact of intervention group for each time interval was determined separately because the *p*‐value for the time interval × intervention group interaction was significant at *α* = 0.1.

In Malawi, the groups did not differ significantly in the mean percentage of days with morbidity symptoms during the follow‐up period. We found a significant intervention group × time interval interaction (*p* = 0.06) for the percentage of days women had severe nasal discharge, but in none of the separate time intervals did the groups differ in this outcome. These findings did not change in either country after adjusting for baseline characteristics (results not shown).

### 3.3 Exploratory analyses

Results of the per‐protocol analysis limiting the analysis to women with high adherence to supplementation (i.e., added supplements to their foods >70% of days during follow‐up) did not differ from those of the main analysis (results not shown). Regarding effect modification, there were some significant interactions between intervention group and certain baseline characteristics, such as maternal age in Ghana and household toilet facility in Malawi, but there were no clear or consistent patterns across time intervals. Given the number of interactions examined, these few significant *P*‐for‐interaction values could have been due to chance.

## 4 DISCUSSION

In Ghana and Malawi, we found that after excluding the first trimester due to the small number of women enrolled in that period, maternal morbidity symptoms were most common in the second trimester of pregnancy and prevalence declined significantly through 3–6 months of lactation. In Ghana, the three intervention groups did not differ significantly in the morbidity outcomes measured, except for the period prevalence of vomiting in the third trimester of pregnancy (greater in the MMN group than the LNS group) and 0–3 months lactation (greater in the MMN group than the IFA group) and mean percentage of days with nausea in the third trimester of pregnancy and during 0–3 months of lactation (greater in the MMN and LNS groups than in the IFA group). Likewise, in Malawi, the three intervention groups differed significantly only in the period prevalence of severe diarrhoea during 3–6 months of lactation (greater in the LNS group than the MMN group). In both countries, the differences between groups in these three outcomes were small and possibly due to chance. Overall, we did not find consistent evidence that SQ‐LNS or MMN increased or decreased maternal morbidity during pregnancy or lactation, compared to IFA.

The strengths of our study included the random allocation of participants to intervention groups and the frequent home visits by study staff to collect the morbidity data. There are potential limitations. First, the self‐reported morbidity data could not be clinically verified and may also be compromised by recall bias. Second, the period prevalence of morbidity outcomes during the second trimester of pregnancy may be underestimates because many women in both countries were enrolled later in that trimester rather than earlier, and thus some morbidity symptoms during that period may have been missed. In Malawi, the biweekly data collection focused on the ‘past 7 days’, which reduced the risk of recall bias (relative to a 14‐day recall period) but meant that we missed collecting morbidity data for approximately one‐half of the days during the follow‐up period. Third, we tested multiple hypotheses involving 11 or 10 morbidity symptoms in either country and therefore, at least some of the observed differences may be due to chance (Li et al., 2017). We did not correct for multiplicity in our hypotheses testing because we considered any such correction as counterproductive (Streiner, 2015) or inappropriate (Leroy et al., 2022; Rothman, 1990; Schulz & Grimes, 2005).

Despite these limitations, self‐reported data may reflect community experiences not often captured in clinic or hospital settings (Kim et al., 2012). These trials included approximately 12 months of maternal follow‐up, which allowed the women to become accustomed to monitoring their own morbidity symptoms, thereby possibly minimising the risk of recall bias. It is also unlikely that any recall bias or possible underestimates of morbidity prevalence in the second trimester of pregnancy would be unique to a specific intervention group or groups.

We reported previously that the intervention groups in Ghana (Adu‐Afarwuah et al., 2015) and Malawi (Ashorn, Alho, Ashorn, Cheung, Dewey, Harjunmaa, et al., 2015) did not differ significantly in the percentage of women who experienced severe adverse events (i.e., hospitalisations, life‐threatening events, deaths, miscarriages, stillbirths, congenital malformations, etc.) during pregnancy. At 36 weeks of pregnancy in Ghana (Adu‐Afarwuah, Lartey, Okronipa, Ashorn, Zeilani et al., 2017), we found no significant group differences in the mean concentrations of two biomarkers of inflammation, C‐reactive protein (CRP) and alpha‐1 glycoprotein (AGP), or the percentage of women with elevated concentrations of these biomarkers. The lack of group differences in most of the morbidity outcomes reported herein is consistent with the previously reported findings.

Morbidity data from other trials evaluating small quantity‐ or medium quantity‐LNS (Dewey et al., 2017; Galasso et al., 2019; Mridha et al., 2016) have not yet been published, such as those from Bangladesh (Dewey et al., 2017; Mridha et al., 2016), in which women received IFA, MMN, or SQ‐LNS during pregnancy or pregnancy and lactation, and in Madagascar (Galasso et al., 2019), in which pregnant and lactating women received 40 g/day LNS but the comparison groups received no supplements. In Nepal (Christian et al., 2009), women receiving folic acid, iron‐folic acid, iron‐folic acid‐zinc, or multiple micronutrients from early pregnancy through 3 months postpartum did not differ in several morbidity outcomes measured during the first 9 days after giving birth, including poor appetite for >2 days, ≥4 watery stools and/or mucus/blood in stools, or persistent cough for >2 days.

In Ghana, the observed group differences in the mean percentage of days of monitoring when women had nausea during the third trimester of pregnancy and 0–3 months postpartum may potentially be related to the perceived metallic ‘taste’ or ‘aftertaste’ of LNS when consumed without being mixed with food (Klevor et al., 2016). However, the finding that the MMN group also reported a greater percentage of days with nausea than the IFA group argues against that potential explanation, given that women swallowed the MMN with water. In Malawi, it is difficult to explain why the period prevalence of severe diarrhoea during 3–6 months postpartum was greater in the LNS group than the MMN group, as there were no differences during any other time interval nor for the mean percentage of days of monitoring when women had severe diarrhoea. These observed differences were small and perhaps not clinically meaningful.

We conclude that Ghanaian and Malawian women receiving IFA, MMN or SQ‐LNS supplements during pregnancy and lactation generally do not appear to differ in morbidity symptoms. Additional studies to examine the impact of SQ‐LNS consumption on maternal morbidity would be useful.

## AUTHOR CONTRIBUTIONS

Seth Adu‐Afarwuah, Anna Lartey, Per Ashorn, Kenneth Maleta and Kathryn G. Dewey designed the research. Seth Adu‐Afarwuah, Anna Lartey and Harriet Okronipa (Ghana) and Per Ashorn, Ulla Ashorn and Kenneth Maleta (Malawi) conducted the research. Seth Adu‐Afarwuah and Charles D. Arnold performed the statistical analysis. Seth Adu‐Afarwuah and Kathryn G. Dewey wrote the manuscript. Anna Lartey, Harriet Okronipa, Kenneth Maleta, Per Ashorn, and Ulla Ashorn reviewed the draft manuscript. All coauthors read and approved the final manuscript.

## CONFLICT OF INTEREST STATEMENT

The authors declare no conflict of interest.

## Data Availability

Data are available from the authors on request.
